# Evaluation of Vaccination Strategies to Compare Efficient and Equitable Vaccine Allocation by Race and Ethnicity Across Time

**DOI:** 10.1001/jamahealthforum.2021.2095

**Published:** 2021-08-20

**Authors:** Patricia Kipnis, Lauren Soltesz, Gabriel J. Escobar, Laura Myers, Vincent X. Liu

**Affiliations:** 1 Division of Research, Kaiser Permanente, Oakland, California; 2 The Permanente Medical Group, Oakland, California

## Abstract

**Importance:**

Identifying the most efficient COVID-19 vaccine allocation strategy may substantially reduce hospitalizations and save lives while ensuring an equitable vaccine distribution.

**Objective:**

To simulate the association of different vaccine allocation strategies with COVID-19–associated morbidity and mortality and their distribution across racial and ethnic groups.

**Design, Setting, and Participants:**

We developed and internally validated the risk of COVID-19 infection and risk of hospitalization models on randomly split training and validation data sets. These were used in a computer simulation study of vaccine prioritization among adult health plan members who were drawn from an integrated health care delivery system. The study was conducted from January 3, 2021, to June 1, 2021, in Oakland, California, and the data were analyzed during the same period.

**Main Outcomes and Measures:**

We simulated the association of different vaccine allocation strategies, including (1) random, (2) a US Centers for Disease Control and Prevention (CDC) proxy, (3) age based, and (4) combinations of models for the risk of adverse outcomes (CRS) and COVID-19 infection (PROVID), with COVID-19-related hospitalizations between May 1, 2020, and December 31, 2020, that were randomly permuted by month across 250 simulations and assessed vaccine allocation by race and ethnicity and the neighborhood deprivation index across time.

**Results:**

The study included 3 202 679 adult patients (mean [SD] age, 48.2 [18.0] years; 1 677 637 women [52.4%]; 1 525 042 men [47.6%]; 611 154 Asian [19.1%], 206 363 Black [6.4%], 642 344 Hispanic [20.1%], and 1 390 638 White individuals [43.4%]), of whom 36 137 (1.1%) were positive for SARS-CoV-2. A risk-based strategy (CRS/PROVID) showed the largest avoidable hospitalization estimates (4954; 95% CI, 3452-5878) followed by age-based (4362; 95% CI, 2866-5175) and CDC proxy (4085; 95% CI, 2805-5109) strategies. Random vaccination showed substantially lower reductions in adverse outcomes. Risk-based strategies also showed the largest number of avoidable COVID-19 deaths (joint CRS/PROVID) and household transmissions. Risk-based (PROVID) and CDC proxy strategies were estimated to vaccinate the highest percentage of Hispanic and Black patients in 8 months (joint CRS/PROVID: 642 570 [100%] Hispanic, 185 530 [90%] Black; PROVID: 642 570 [100%] Hispanic, 198 480 [96%] Black; CDC proxy: 605 770 [95%] Hispanic and 151 772 [74%] Black) compared with an age-based approach (438 423 [68%] Hispanic, 154 714 [75%] Black). Overall, the PROVID and joint CRS/PROVID risk-based strategies were estimated to be followed by the most patients from areas with high neighborhood deprivation index being vaccinated early.

**Conclusions and Relevance:**

In this simulation modeling study of adults from a large integrated health care delivery system, risk-based strategies were associated with the largest estimated reductions in COVID-19 hospitalizations, deaths, and household transmissions compared with the CDC proxy and age-based strategies, with a higher proportion of Hispanic and Black patients were estimated to be vaccinated early in the process compared with the CDC strategy.

## Key Points

**Question:**

What is the association of different vaccine allocation strategies with COVID-19–related morbidity and mortality and their distribution by racial and ethnic groups across time?

**Findings:**

In this decision analytical model, the use of risk-based, age-based, and US Centers for Disease Control and Prevention (CDC)–phased vaccine allocation strategies was simulated. Risk-based strategies were associated with the largest estimated reductions in nonelective hospitalizations, death, and household transmissions compared with the CDC- and age-based strategies, with a similar proportion of Hispanic and Black patients being vaccinated early in the process compared with the CDC strategy.

**Meaning:**

The study findings suggest that risk-based vaccine prioritization strategies could have the greatest effectiveness on reducing COVID-19–related deaths and household transmissions while ensuring equitable vaccine distribution.

## Teaser

This decision analytical model simulates the association of different vaccine allocation strategies with COVID-19–associated morbidity and mortality and their distribution across racial and ethnic groups.

## Introduction

SARS-CoV-2 infection and COVID-19 disease have resulted in more than 22 million US COVID-19 cases and 400 000 deaths,^1^ severely straining hospitals, disrupting communities, amplifying social disparities, and contributing to social unrest worldwide.^2,3,4,5,6^ Vaccination remains the key to controlling the COVID-19 disease,^7^ with the recent development of effective COVID-19 vaccines representing a critical step forward.^8,9^ Given the limitations in vaccine supply in early 2021, the Advisory Committee on Immunization Practices (ACIP) recommended phased COVID-19 vaccine allocation in the US^10,11^ (eTables 1-5 in the Supplement).

While the ACIP allocation hierarchy sought to balance exposure risk factors, morbidity, and mortality along with social and equity priorities, implementing this strategy proved challenging, with many states pivoting to alternate approaches like age-based stratification. A key limitation has been the availability of data needed to implement the ACIP guidelines.^12,13^ While routine electronic health record (EHR) data could be used to inform vaccination, to our knowledge few studies have evaluated their potential role in improving allocation, particularly under inadequate vaccine supply constraints. In this study, we used EHR data from an integrated health care delivery system to simulate the potential association of different vaccine allocation strategies with estimated COVID-19–related morbidity, mortality, household transmission, and racial and ethnic group vaccine distribution.

## Methods

This computer simulation study adheres to the health economic modeling report guidelines (Consolidated Health Economic Evaluation Reporting Standards; eTable 7 in the Supplement).^14^ Kaiser Permanente Northern California (KPNC) is an integrated health care delivery system that serves 4.5 million members. This project was approved by the KPNC institutional review board, which waived the requirement for individual informed consent because of the large number of study participants.

### Study Population

The overall cohort included adult (≥18 years) KPNC patients with continuous membership or utilization (unless they died) between February 1, 2020 and December 3, 2020. We captured members’ self-reported race and ethnicity (documented at the time of insurance enrollment by medical assistants in the outpatient setting and/or admission clerks for inpatients); comorbidities^15,16,17^; and neighborhood deprivation index (NDI), a composite index ranging from −5 to +5, with more positive values indicating worsening socioeconomic status (eg, poverty and unemployment).^18^ We also calculated 2 previously validated general illness risk-adjustment indices: the Comorbidity Point Score, version 2 (COPS2), to assess 1-year comorbid disease burden and the abbreviated Laboratory-Based Acute Physiology Score (abLAPS), which is an outpatient laboratory-based severity of illness score (eAppendix in the Supplement).^19,20^

### COVID-19 Risk Scores

Between the first 2 COVID-19 waves in Northern California, we developed 2 scores to classify patients within COVID-19 risk groups using data from February 1, 2020, to September 30, 2020: (1) the PROVID (probability of COVID-19 infection) to measure the likelihood of a patient having a positive SARS-CoV-2 polymerase chain reaction test result and (2) the COVID-19 Risk Score (CRS) to measure the risk of adverse outcomes (ie, hospitalization, death) among patients with COVID-19.^21^ The PROVID score used a logistic regression model with a positive SARS-CoV-2 test result as the outcome and predictors of age, sex, race and ethnicity,^22^ COPS2, abLAPS, and NDI. The model was fitted on a random training set (70% of the data) and validated on the remaining 30%, with a validation area under the receiver operating characteristic curve of 0.71 (95% CI, 0.71-0.72). The CRS score quantified the risk of nonelective hospitalization within 30 days of a SARS-CoV-2 positive test result using a logistic regression model for patients with COVID-19 that included sex and truncated power function splines at 3 selected knots^23^ for age and COPS2.^21^ We fitted the CRS model on 75% of the data and evaluated its performance on the remaining 25%. The model had a validation area under the curve of 0.82 (95% CI, 0.81-0.83) for 30-day nonelective hospitalization.

We calculated CRS and PROVID scores for all adult KPNC members in the cohort and divided patients into deciles establishing low- (<40th percentile), medium- (40th to 60th percentile), and high-risk (>60th percentile) groups for each score. We characterized the demographic, clinical, and hospital utilization characteristics of the 9 joint CRS-PROVID risk groups based on the interactions between the 3 CRS and 3 PROVID risk strata (eTables 1-5 in the Supplement).

### Outcomes

Because the intent was to explore the association of vaccination strategies with adverse outcomes from a public health perspective, our primary outcomes were COVID-19 hospitalizations, deaths, and household transmissions. A hospitalization was classified as COVID-19 related if the physician designation during hospital treatment was either *confirmed active COVID* or *resolved COVID*. COVID-19 deaths were inpatient deaths that occurred during COVID-19–related hospitalizations. To estimate COVID-19 household transmission, we grouped KPNC members into households according to their address in EHR data. In each household, we identified the first member with a positive SARS-CoV-2 test result and quantified COVID-19 transmission within a household based on the number of household members who tested positive within 4 weeks of that first household positive test result. As secondary outcomes, we evaluated the rate of vaccination by NDI and among 4 broad racial and ethnic groups (White, Black and African American, Hispanic, and Asian individuals).

### Vaccination Strategy Simulation

The detailed data sources at KPNC allowed us to conduct a computer simulation study which is easier to understand and requires fewer modeling assumptions compared with a mathematical simulation model. We simulated 6 vaccination strategies assuming a target goal of vaccinating 75% of the 3 million adult KPNC members (2 400 000) over an 8-month period (240 days) with a uniform vaccination rate of 10 000 vaccines administered daily. When priority groups were simulated, we assumed that patients within the same priority grouping were vaccinated in random order and that all group members would be vaccinated before moving to the next group. Finally, we assumed that all phase 1a–eligible patients (ie, health care personnel and long-term care facility residents) completed vaccination before each prioritization strategy began. Four strategies were used. The first was a random vaccine allocation, which was a baseline process with randomly distributed vaccinations. The second was a CDC proxy, which was an ACIP-based process starting with essential workers, defined as patients ages 18 to 65 years with a PROVID score greater than the 80th percentile (1.6%) based on the most recent 5-year estimates from the American Community Survey (2014-2018).^24,25^ We defined patients with high-risk medical conditions (phase 1c) as those with a CRS score of 20% or greater, as used at KPNC to define higher-risk patients for COVID-19–related interventions. The remaining essential workers were defined as those with a PROVID in the 50th to 80th percentile (0.71%-1.6%). If patients qualified for several vaccination phases, we assigned them to their highest qualified tier (eTable 1 in the Supplement). The third strategy was age based and was developed around age groups in reverse order (age ≥75, 65-74, 55-64, 45-54, 35-44, and 18-34 years). The fourth strategy was risk based and involved a process in which vaccination prioritization was determined by the rate of hospitalizations across the 9 joint CRS/PROVID risk groups, starting with the high PROVID/high CRS group and ending with the low CRS/low PROVID group. We also assessed vaccine prioritization strategies by either risk score alone (PROVID or CRS alone).

### Statistical Analyses

We calculated the number of outcomes during the 8-month period of May 1, 2020, through December 31, 2020, among patients that corresponded to each strategy until 2.4 million patients were vaccinated and compared the age-based, CDC proxy, and risk-based strategies with random vaccination order. To estimate the association between allocation strategies and outcomes, we conducted 250 simulations in which we permuted the month of COVID-19–related hospitalization month to simulate random monthly outcome distributions over the vaccination period. We assumed that any vaccination would be 100% effective at preventing hospitalization; thus, we removed hospitalizations that occurred at any time after 1 month from the vaccination date. In each simulation, we calculated the difference between the actual and simulated number of hospitalizations to estimate the number of avoided hospitalizations. We reported the average difference in hospitalizations and deaths across the 250 simulations and the 2.5th and 97.5th percentile as the 95% confidence interval. We followed a similar strategy to simulate SARS-CoV-2 transmissions within households. We permuted the first COVID-19 household test month in 250 simulations and assumed that vaccination would prevent transmission for transmissions that occurred within 2 weeks of receiving the vaccine. Finally, we compared the percentage of vaccinated patients by race and ethnicity and NDI (>75th percentile) to assess equitable distribution of the vaccine within each allocation strategy. Statistical analyses were conducted using SAS, version 7.15 (SAS Institute).

### Sensitivity Analysis

We also varied the underlying age and race distribution of the population based on US census data^24^ to identify the individual states with the youngest (470 905 [14.7%] younger than age 65 years); oldest (804 063 [25.1%] older than age 65 years); highest percentage Asian (1 220 509 [38.1%]), African American (1 412 716 [44.1%]), Hispanic (1 579 294 [49.3%]) or White (2 972 790 [92.8%] populations); and lowest percentage African American (19 221 [0.6%]) and Hispanic (48 052 [1.5%]) populations (eTable 8 in the Supplement). We then created bootstrap samples of the KPNC data with the corresponding age and race distributions of these states and simulated the association between the vaccine prioritization strategies and adverse outcomes among populations of KPNC patients with these characteristics.

## Results

We identified 3 202 679 adults, including 36 137 (1.1%) with positive SARS-CoV-2 test results (Table 1^18,19,26^). Patients who tested positive for SARS-CoV-2 during the study period tended to be younger (mean [SD] age, 43.4 [16.3] vs 48.2 [18.0] years; *P* < .001), had fewer comorbidities (COPS2 of 14.2 vs 14.7; *P* < .001), and were less likely to be White (23% vs 44%; *P* < .001) compared with those who tested negative or without a test. Among those testing positive, 3403 (9.4%) experienced nonelective hospitalization within 30 days of their test. Hospitalized patients with COVID-19 were older (mean [SD] age, 59.5 [17.1] vs 41.8 [15.3] years; *P* < .001), had more comorbidities (mean COPS2, 30.4 vs 12.6; *P* < .001) and had more acute illness (mean abLAPS, 1.8 vs 0.3; *P* < .001) than those not hospitalized.

**Table 1.** Characteristics of Kaiser Foundation Health Plan Adult Members as of February 1, 2020

Variable	No. (%)				
	All adults			Adults with positive test result	
	Total	No positive test result	With positive test result	No hospitalization	Hospitalization^a^
No.	3 202 679	3 166 542 (98.9)	36 137 (1.1)	32 734 (90.6)	3403 (9.4)
Mean (SD) age, y	48.2 (18.0)	48.2 (18.0)	43.4 (16.3)	41.8 (15.3)	59.5 (17.1)
Women	1 677 637 (52.4)	1 658 668 (52.4)	18 969 (52.5)	17 403 (53.2)	1566 (46.0)
Men	1 525 042 (47.6)	1 507 874 (47.6)	17 168 (47.5)	15 331 (46.8)	1837 (54.0)
NDI^b^	−0.3 (0.9)	−0.3 (0.9)	0.2 (0.9)	0.2 (0.9)	0.2 (1.0)
Race and ethnicity					
Asian	611 154 (19.1)	606 815 (19.2)	4339 (12.0)	3834 (11.7)	505 (14.8)
Black	206 363 (6.4)	203 849 (6.4)	2514 (7.0)	2166 (6.6)	348 (10.2)
Hispanic	642 344 (20.1)	624 552 (19.7)	17 792 (49.2)	16 329 (49.9)	1463 (43.0)
Other^c^	352 180 (11.0)	348 945 (11.0)	3235 (9.0)	2996 (9.2)	239 (7.0)
White	1 390 638 (43.4)	1 382 381 (43.7)	8257 (22.8)	7409 (22.6)	848 (24.9)
abLAPS^d^	0.4 (3.0)	0.4 (3.0)	0.5 (3.2)	0.3 (2.4)	1.8 (7.2)
COPS2^e^	14.7 (17.1)	14.7 (17.1)	14.2 (17.8)	12.6 (13.1)	30.4 (37.7)
Charlson Comorbidity Score	0.5 (1.3)	0.5 (1.3)	0.5 (1.3)	0.4 (1.0)	1.7 (2.3)
PROVID risk group					
Low (0.3%-0.63%)	1 281 070 (40.0)	1 275 398 (40.3)	5672 (15.7)	4841 (14.8)	831 (24.4)
Medium (0.63%-0.99%)	960 805 (30.0)	952 805 (30.1)	8000 (22.1)	7290 (22.3)	710 (20.9)
High (0.99%-18%)	960 804 (30.0)	938 339 (29.6)	22 465 (62.2)	20 603 (62.9)	1862 (54.7)
CRS risk group					
Low (0.7%-4.9%)	1 263 178 (39.4)	1 245 658 (39.3)	17 520 (48.5)	17 045 (52.1)	475 (14.0)
Medium (5%-10.3%)	973 608 (30.4)	962 408 (30.4)	11 200 (31.0)	10 274 (31.4)	926 (27.2)
High (10.4%-100%)	965 893 (30.2)	958 476 (30.3)	7417 (20.5)	5415 (16.5)	2002 (58.8)

Abbreviations: abLAPS, abbreviated laboratory-based acute physiology score; COPS2, Comorbidity Points Score 2; CRS, COVID-19 risk score; NDI, neighborhood deprivation index; PROVID, probability of COVID-19 infection.

^a^ Within 30 days of first test.

^b^ See article and Messer et al^18^ for additional details on the NDI; this index ranges between −5 to +5, with more positive values indicating worsening neighborhood characteristics (eg, poverty, unemployment). Number shown is median (interquartile range).

^c^ Analysis by race and ethnicity was limited to Asian, Black, Hispanic, and White race and ethnicity. “Other” race category includes Pacific Islander, American Indian, Alaska Native, and multiracial individuals.

^d^ The abLAPS score is a monthly score that uses 14 laboratory tests based on the LAPS score described in Escobar et al.^19^ The range is from 0 to 256; higher scores indicate increasing physiologic abnormalities during the preceding month. In recent internal analyses, the univariate relationship between the abLAPS and 30-day mortality is as follows: 0 to 4, 0.06%; 4 to 9, 0.18%; 10 or greater, 1.32%.

^e^ The COPS2 score, described in Escobar et al,^26^ is a score assigned every month to all adults with a Kaiser Permanente Northern California medical record number. The range is from 0 to 1010; higher scores indicate worse mortality risk. The univariate association between the COPS2 and 1-year mortality is as follows: 0 to 39, 0.3%; 40 to 64, 5.3%; 65 or greater, 17.2%.

Patients with a high risk of SARS-CoV-2 infection (higher PROVID scores) were more likely to be Hispanic (60%-70% vs <5%; *P* < .001 for all comparisons) and Black (12%-16% vs 0%-9%), to test positive for COVID-19 (20-24 vs 4-9 per 1000), and to have higher mean NDI scores (0.4 to 0.5 vs −1.1 to −0.2). Patients with high risk of adverse outcomes (higher CRS scores) were older (age 65-70 years vs 30-50 years; *P* < .001) and more likely to be male (60% vs 40%-50%; *P* < .001) than those with low and medium risk (eTable 6 and eFigures 1-4 in the Supplement).

The oldest patients (75 years or older; n = 262 005) represented only 8.2% of the population but accounted for 25.4% of COVID-19 hospitalizations and 50.6% of inpatient deaths. In contrast, patients aged 18 to 54 years (n = 1 993 356) represented 62.2% of members, 34.4% of COVID-19 hospitalizations, and 11.3% of inpatient deaths (Table 2). Patients in the joint high CRS/high PROVID risk group had the highest hospitalization rate (12.1 per 1000 members), followed by those in the high CRS/medium PROVID risk group (6.8 per 1000 members). These 2 groups represented 11% of the population (n = 347 706) and accounted for 38.6% of all COVID-19 hospitalizations and 53.2% of inpatient deaths. Patients in the high PROVID/medium CRS and low PROVID/high CRS represented 28.2% of the population (n = 904 931) and accounted for another 38.6% of the COVID-19 hospitalizations and 42.1% of inpatient deaths.

**Table 2.** COVID-19 Vaccine Allocation Strategies

Classification group	PROVID	CRS	No. in the population (%)	Mean No. vaccinated (% vaccinated in 8 mo)	COVID-19 hospitalizations per 1000 members	Percentage of total COVID-19 hospitalizations	Inpatient deaths per 100 000 members	Percentage of deaths
All adults	NA	NA	3 203 437 (100)	2 400 000 (74.9)	2.7	100	24.1	100
CDC proxy								
Essential (high PROVID)	NA	NA	566 539 (17.7)	566 539 (100)	4.7	31.4	30.2	22.2
Age ≥75 y			253 409 (7.9)	253 409 (100)	7.7	22.9	138.5	45.5
Age 65-74 y			375 612 (11.7)	375 612 (100.0)	3.5	15.3	33.8	16.5
High CRS			72 729 (2.3)	72 729 (100)	7.0	6.0	46.7	4.4
Essential (mid PROVID)			849 696 (26.5)	849 696 (100)	1.1	11.1	4.0	4.4
All others^a^			1 085 452 (33.9)	282 015 (26.0)	1.0	13.3	5.1	7.1
Age-based, y								
≥75	NA	NA	262 005 (8.2)	262 005 (100)	8.2	25.4	149.2	50.6
65-74			398 693 (12.4)	398 693 (100)	4.1	19.3	41.6	21.5
55-64			549 383 (17.1)	549 383 (100)	3.2	20.8	23.3	16.6
45-54			543 360 (17.0)	543 360 (100)	2.5	15.8	11.0	7.8
35-44			575 337 (18.0)	575 337 (100)	1.5	9.9	3.1	2.3
18-34			874 659 (27.3)	71 222 (8.1)	0.8	8.7	1.0	1.2
Risk-based	High	High	175 943 (5.5)	175 943 (100)	12.1	24.9	146.6	33.4
	Medium	High	171 763 (5.4)	171 763 (100)	6.8	13.6	89.1	19.8
	High	Medium	286 139 (8.9)	286 139 (100)	4.1	13.8	17.1	6.3
	Low	High	618 792 (19.3)	618 792 (100.0)	3.4	24.8	44.6	35.8
	High	Low	499 065 (15.6)	499 065 (100)	1.6	9.6	1.4	0.9
	Medium	Medium	296 371 (9.3)	296 371 (100)	1.6	5.6	5.4	2.1
	Low	Medium	391 202 (12.2)	351 927 (90.0)	0.8	3.5	2.0	1.0
	Medium	Low	492 840 (15.4)	0	0.6	3.5	0.8	0.5
	Low	Low	271 322 (8.5)	0	0.2	0.5	0.4	0.1

Abbreviations: CDC, US Centers for Disease Control and Prevention; CRS, COVID-19 Risk Score; PROVID, probability of COVID-19 infection.

^a^ All remaining patients 18 years or older.

**Figure 1.**
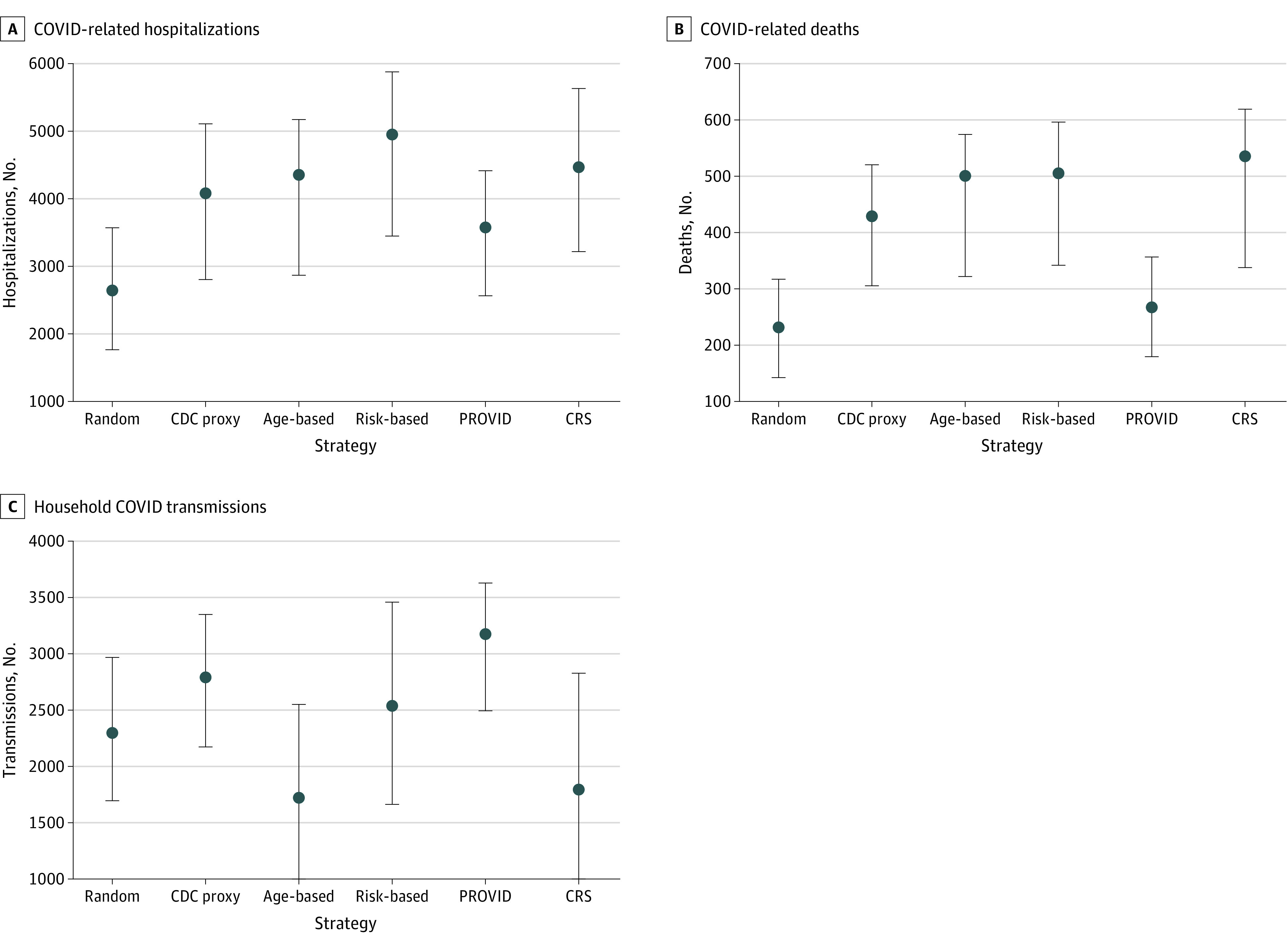
Estimated Avoidable Hospitalizations, Deaths, and Household COVID-19 Transmissions Among Patients Vaccinated in First 8 Months by Vaccination Prioritization Order There were 7867 COVID-19 hospitalizations, 675 inpatient deaths, and 8712 within-household COVID-19 transmissions from May to December 2020. Error bars indicate 95% CIs. CDC indicates US Centers for Disease Control and Prevention; CRS, COVID-19 risk score; PROVID, probability of COVID-19 infection.

Over the 8-month study period, there were 7867 COVID-19 hospitalizations and 675 inpatient deaths. The simulated random allocation strategy was associated with estimates of 2645 (95% CI, 1766-3574; Figure 1) avoidable hospitalizations and 232 (95% CI, 143-317) avoidable inpatient deaths. Comparatively, the joint CRS/PROVID and CRS risk-based vaccination strategies were associated with the largest estimates of avoidable COVID-19 hospitalizations (CRS/PROVID: 4954; 95% CI, 3452-5878; CRS: 4470; 95% CI, 3215-5631) and deaths (CRS/PROVID: 505; 95% CI, 343-596; CRS: 535; 95% CI, 338-619). The age-based and CDC proxy approaches also showed higher estimated avoidable hospitalizations and deaths than random allocation (age-based: 4362; 95% CI, 2866-5175; CDC proxy: 4085; 95% CI, 2805-5109).

The random allocation strategy was associated with an estimated 2301 (95% CI, 1696-2967) household transmissions prevented. A PROVID-based risk approach showed the highest estimated avoidable household transmissions (3176; 95% CI, 2496-3632), followed by the CDC proxy (2793; 95% CI, 2173-3352), the joint CRS/PROVID (2538; 95% CI, 1663-3460), the CRS (1796; 95% CI, 882-2832), and age-based (1722; 95% CI, 892-2549) strategies. In sensitivity analyses, results were similar when varying the age and race and ethnicity distributions, with the joint CRS/PROVID risk-based strategy showing the largest estimated reduction in hospitalizations and deaths (eTable 6 and eFigures 1-4 in the Supplement).

**Figure 2.**
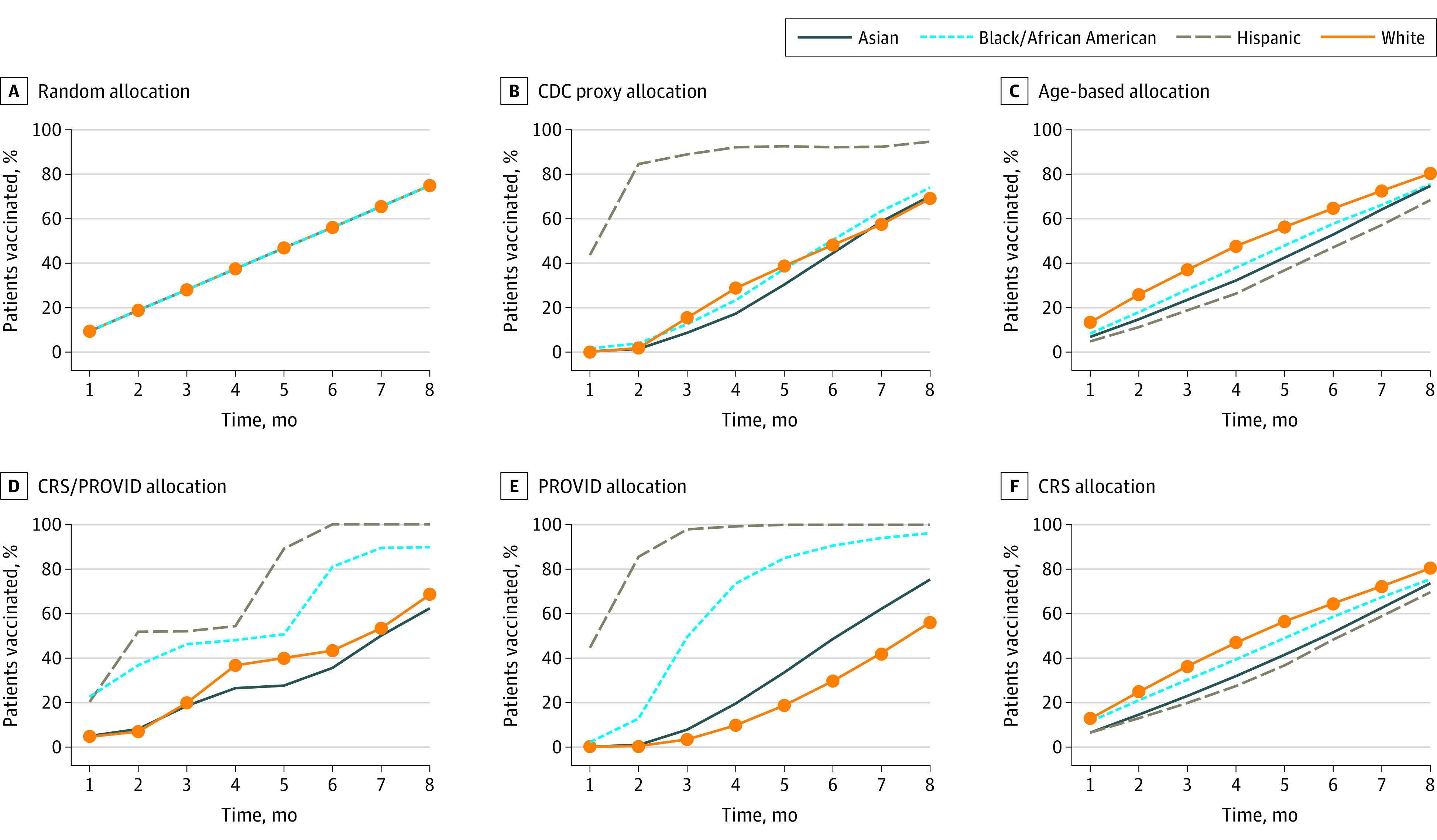
Cumulative Percentage of Patients Vaccinated by Race, Vaccination Month, and Strategy CRS indicates COVID-19 risk score; PROVID, probability of COVID-19 infection.

**Figure 3.**
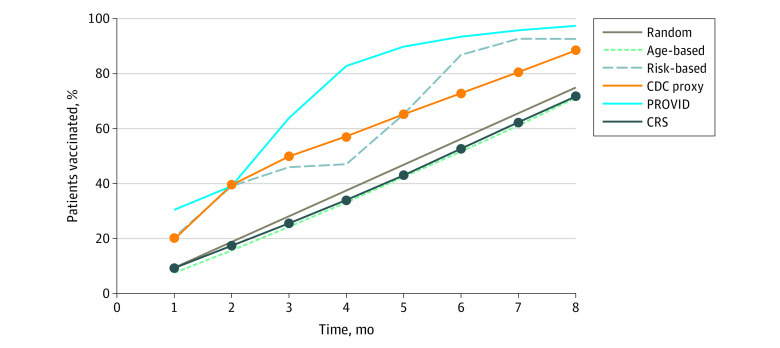
Percentage of Total Vaccinated With High Neighborhood Depravation Index (NDI) by Vaccination Month and Strategy High NDI is defined as an NDI at or greater than the 75th percentile of all adults in the study. CRS indicates COVID-19 risk score; PROVID, probability of COVID-19 infection.

The risk-based joint CRS/PROVID, PROVID risk allocation, and CDC proxy strategies vaccinated the highest percentage of Hispanic and Black patients in 8 months (CRS/PROVID: 642 570 [100%] Hispanic, 185 530 [90%] Black; PROVID: 642 570 [100%] Hispanic, 198 480 [96%] Black; CDC proxy: 605 770 [95%] Hispanic and 151 772 [74%] Black) compared with an age-based approach (438 423 [68%] Hispanic, 154 714 [75%] Black; Figure 2). Overall, the PROVID and CRS/PROVID risk-based strategies resulted in the most patients from areas with high neighborhood deprivation being vaccinated early (Figure 3).

## Discussion

Using data from a large, diverse, and integrated health care delivery system, we simulated the implementation of different COVID-19 vaccine allocation strategies and compared their association with estimates of vaccine allocation effectiveness and equity. We found that risk-based strategies that identified patients at high risk for adverse outcomes were associated with the highest estimates of avoidable hospital admissions and deaths, followed by the CDC proxy and age-based strategies. We also found that a risk-based approach that identified patients at high risk for COVID-19 infection, followed by the CDC proxy strategy, achieved the goal of earlier vaccinations being administered to Hispanic and Black patients. Simulating the association of these differing prioritization strategies with adverse outcomes could help identify and mitigate some of the potential racial and ethnic and socioeconomic inequities that have been potentiated during the COVID-19 pandemic. Finally, we found that a risk-based allocation strategy that prioritized the risk of infection was associated with the highest estimates of avoidable household transmission, followed by the CDC proxy and joint risk-based approach. Overall, our analyses suggest that risk-based strategies using available EHR data could inform optimal approaches for improving vaccination effectiveness, efficiency, and equity.

Recent studies on COVID-19 vaccine distribution have focused on policies and practices to support the equity, transparency, accountability, availability, and access to COVID-19 vaccines.^27^ While Schmidt et al^28^ compared different US approaches for implementing the CDC vaccine distribution recommendations to reduce inequity, other studies have highlighted the challenges of implementing the CDC strategy. These include its complexity, political considerations, and equity.^12,29,30^ Mathematical modeling studies also showed similar findings that prioritizing older adults could lead to reduced adverse outcomes,^31,32^ while alternate strategies that target younger adults, who are more likely to be essential workers or have higher infection potential, could reduce overall transmission.^33,34^ Another recent study compared the efficacy of risk-based, age-based, and CDC-phased vaccine allocation approaches to reduce COVID-19-related deaths^35^; however, the risk-based approach was based on only a single model, the CDC approach was defined by age alone, and the study did not evaluate other salient outcomes.

Concerns about the equity of vaccination approaches have been confirmed, as emerging data revealed racial and ethnic inequities in vaccine allocation.^12^ For example, as of March 1, 2021, and including data from 41 states, there was consistent evidence of Hispanic and Black patients receiving smaller shares of vaccines compared with their rates of infection and hospitalization.^36^ In some locales, Asian patients, who also have increased rates of infection compared with White patients, also had lower relative rates of vaccination.^22^ While concerns have been raised about the legal implications of vaccine allocation by race and ethnicity, legally permissible strategies could use NDI, which integrates income levels, education, employment, and housing quality, to address vaccination.^37^ However, the urgent need to understand and simulate the vaccine allocation process and its resulting effect on marginalized patient groups remains.^6,38,39^

While the US is entering a period of decreasing vaccination rates, vaccine supply remains highly constrained across the world today. Thus, the estimates produced in this simulation can help inform vaccine allocation approaches in locales with limited vaccine supply or potentially in future periods of vaccine distribution for COVID-19 or other similar pandemics. In addition, risk-based approaches could be used to inform public health and community engagement campaigns that are designed to increase vaccination uptake among groups that remain undervaccinated.

### Strengths and Limitations

The major strength of this study was the ability to find an association between detailed population-level data with SARS-CoV-2 test results and COVID-19 hospital admissions, deaths, and household transmission through facilitating the use of 2 risk models and simulating key outcomes. The study also quantifies the effectiveness and equity of these approaches in a large and diverse patient population. This was possible because of the high degree of integration at KPHC and because it has made a concerted effort to capture race and ethnicity data in the outpatient setting.

Our study also has important limitations. First, the CDC vaccine allocation process we simulated is only a proxy because we lacked access to high-quality information about essential workers’ status; however, the risk scores we developed could be valuable as a proxy to other health systems that similarly lack employment data Second, we also used prior COVID-19 hospitalization data, those before the availability of any vaccine outside of clinical trials, to understand the association of vaccination allocation and future hospitalizations. This assumed that the association of future surges on hospitalization and death would be similar to what we have already observed. While future COVID-19 sequelae might differ from that observed in 2020, we compared all strategies using the same assumptions and hospitalization data. Third, we did not simulate a scenario in which vaccinated patients were less likely to transmit the virus.^40^ Fourth, we did not consider deaths associated with the vaccine, which have proven to be extremely rare.^41^ Fifth, we did not consider different vaccine uptake levels among different racial and ethnic groups.^42^ Sixth, our estimates of household transmission should be viewed as provisory, as they likely reflect differential testing patterns and characteristics across households. Finally, the simulation does not consider any differential efficacy or reduction in the efficacy of differing vaccines over time.

## Conclusions

In this simulated modeling study of adults from a large integrated health care delivery system, risk-based strategies were associated with the largest estimated reductions in COVID-19 hospitalizations, deaths, and household transmissions compared with the CDC proxy and age-based strategies, with similar proportions of Hispanic and Black patients being vaccinated early in the process compared with the CDC strategy.

## Notes

Supplement. **eTable 1.** Implementation of the Advisory Committee on Immunization Practices (ACIP) guidelines **eTable 2.** Probability of COVID-19 Infection (PROVID) Model Coefficients **eTable 3.** COVID-19 Risk Score (CRS) Model Coefficients **eTable 4. **Probability of COVID-19 Infection (PROVID) Deciles **eTable 5. **COVID-19 Risk Score (CRS) Deciles **eAppendix. **Calculation of the Abbreviated Laboratory-Based Acute Physiology Score (abLAPS) **eTable 6. **Characteristics of Patients by Probability of COVID-19 Infection (PROVID) and COVID-19 Risk Score (CRS) Levels **eFigure 1. **Estimated Avoidable Hospitalizations among Patients Vaccinated in First 8 Months by Vaccination Prioritization Order in Populations with Varying Age and Race Distributions **eFigure 2. **Estimated Avoidable Household COVID-19 Deaths among Patients Vaccinated in First 8 Months by Vaccination Prioritization Order in Populations with Varying Age and Race Distributions **eFigure 3. **Estimated Avoidable Household COVID-19 Transmissions among Patients Vaccinated in First 8 Months by Vaccination Prioritization Order in Populations with Varying Age and Race Distributions **eFigure 4. **Cumulative Percent of Patients Vaccinated by Race, Vaccination Month and Strategy in Populations with Varying Age and Race Distributions **eTable 7. **Consolidated health economic evaluation reporting standards (CHEERS) statement checklist **eTable 8. **Age and Race/Ethnicity Distribution across U.S. States
