# Supplementary material for: Evaluation of Vaccination Strategies to Compare Efficient and Equitable Vaccine Allocation by Race and Ethnicity Across Time
Source: JAMA Health Forum. 2021 Aug 20;2(8):e212095. doi: 10.1001/jamahealthforum.2021.2095 (PMC8796992; doi:10.1001/jamahealthforum.2021.2095)
Supplement: Supplement. — eTable 1. Implementation of the Advisory Committee on Immunization Practices (ACIP) guidelines eTable 2. Probability of COVID-19 Infection (PROVID) Model Coefficients eTable 3. COVID-19 Risk Score (CRS) Model Coefficients eTable 4. Probability of COVID-19 Infection (PROVID) Deciles eTable 5. COVID-19 Risk Score (CRS) Deciles eAppendix. Calculation of the Abbreviated Laboratory-Based Acute Physiology Score (abLAPS) eTable 6. Characteristics of Patients by Probability of COVID-19 Infection (PROVID) and COVID-19 Risk Score (CRS) Levels eFigure 1. Estimated Avoidable Hospitalizations among Patients Vaccinated in First 8 Months by Vaccination Prioritization Order in Populations with Varying Age and Race Distributions eFigure 2. Estimated Avoidable Household COVID-19 Deaths among Patients Vaccinated in First 8 Months by Vaccination Prioritization Order in Populations with Varying Age and Race Distributions eFigure 3. Estimated Avoidable Household COVID-19 Transmissions among Patients Vaccinated in First 8 Months by Vaccination Prioritization Order in Populations with Varying Age and Race Distributions eFigure 4. Cumulative Percent of Patients Vaccinated by Race, Vaccination Month and Strategy in Populations with Varying Age and Race Distributions eTable 7. Consolidated health economic evaluation reporting standards (CHEERS) statement checklist eTable 8. Age and Race/Ethnicity Distribution across U.S. States [file jamahealthforum-e212095-s001.pdf]

Supplemental Online Content

Kipnis P, Soltesz L, Escobar GJ, Myers L, Liu VX. Evaluation of vaccination strategies to compare efficient and equitable vaccine allocation by race and ethnicity across time. *JAMA Health Forum*. 2021;2(8):e212095. doi:10.1001/jamahealthforum.2021.2095

eTable 1. Implementation of the Advisory Committee on Immunization Practices (ACIP) guidelines
eTable 2. Probability of COVID-19 Infection (PROVID) Model Coefficients
eTable 3. COVID-19 Risk Score (CRS) Model Coefficients
eTable 4. Probability of COVID-19 Infection (PROVID) Deciles
eTable 5. COVID-19 Risk Score (CRS) Deciles
eAppendix. Calculation of the Abbreviated Laboratory-Based Acute Physiology Score (abLAPS)
eTable 6. Characteristics of Patients by Probability of COVID-19 Infection (PROVID) and COVID-19 Risk Score (CRS) Levels
eFigure 1. Estimated Avoidable Hospitalizations among Patients Vaccinated in First 8 Months by Vaccination Prioritization Order in Populations with Varying Age and Race Distributions
eFigure 2. Estimated Avoidable Household COVID-19 Deaths among Patients Vaccinated in First 8 Months by Vaccination Prioritization Order in Populations with Varying Age and Race Distributions
eFigure 3. Estimated Avoidable Household COVID-19 Transmissions among Patients Vaccinated in First 8 Months by Vaccination Prioritization Order in Populations with Varying Age and Race Distributions
eFigure 4. Cumulative Percent of Patients Vaccinated by Race, Vaccination Month and Strategy in Populations with Varying Age and Race Distributions
eTable 7. Consolidated health economic evaluation reporting standards (CHEERS) statement checklist
eTable 8. Age and Race/Ethnicity Distribution across U.S. States

This supplemental material has been provided by the authors to give readers additional information about their work.

© 2021 Kipnis P et al. *JAMA Health Forum.*

eTable 1. Implementation of the Advisory Committee on Immunization Practices (ACIP) guidelines[7]

Phase	Groups recommended to receive COVID-19 vaccine	Proxy
1a	Health care personnel	NA
	Long-term care facility residents	NA
1b	Frontline essential workers§	Ages 18-64 and PROVID >80th percentile (1.6%)
	Persons aged ≥75 years	
1c	Persons aged 65–74 years	
	Persons aged 16–64 years with high-risk medical conditions	CRS $\geq$ 20%
	Essential workers not recommended for vaccination in Phase 1b	PROVID in the 50th to 80th percentile (0.71% to 1.6%)
2	All persons aged ≥16 years not previously recommended for vaccination	

eTable 2. Probability of COVID-19 Infection (PROVID) Model Coefficients

**Predictor**	**Coefficient**
Intercept	-5.0322
Male	-0.0138
Age	0.0249
Age^2	-0.0004
COPS2	-0.0105
$COPS2^2$	-0.000018
abLAPS	0.0196
$abLAPS^2$	-0.0000004
Age* abLAPS	-0.0003
Age*COPS2	0.0003
abLAPS*COPS2	-0.0000059
Neighborhood deprivation index	0.3528
Black/African American	0.3219
Hispanic	1.1511
Other Race	0.1594
White	-0.1084

© 2021 Kipnis P et al. *JAMA Health Forum.*

eTable 3. COVID-19 Risk Score (CRS) Model Coefficients

Predictor	Coefficient
Intercept	-6.24629
COPS2	0.21373
$COPS2^2$	-0.04148
$COPS2^3$	0.00193
$max(0,(COPS2-10))^3$	-0.00259
$max(0,(COPS2-20))^3$	0.00069
$max(0,(COPS2-50))^3$	-3.3E-05
Age	0.05617
Age^2	0.00172
Age^3	-3E-05
$max(0,(Age-30))^3$	3.3E-05
$max(0,(Age-50))^3$	2.1E-05
$max(0,(Age-65))^3$	-4.9E-05
Male	0.34514

© 2021 Kipnis P et al. *JAMA Health Forum.*

eTable 4. Probability of COVID-19 Infection (PROVID) Deciles

PROVID Decile	N	Mean	Minimum	Maximum
1	320,267	0.003	0.000	0.004
2	320,268	0.005	0.004	0.005
3	320,268	0.005	0.005	0.006
4	320,267	0.006	0.006	0.006
5	320,269	0.007	0.006	0.007
6	320,266	0.008	0.007	0.008
7	320,270	0.009	0.008	0.010
8	320,268	0.013	0.010	0.017
9	320,268	0.021	0.017	0.026
10	320,268	0.036	0.026	0.179

© 2021 Kipnis P et al. *JAMA Health Forum.*

eTable 5. COVID-19 Risk Score (CRS) Deciles

CRS Decile	N	Mean	Minimum	Maximum
1	311,451	0.01	0.01	0.02
2	282,912	0.02	0.02	0.02
3	339,386	0.03	0.02	0.04
4	329,429	0.04	0.04	0.05
5	337,460	0.06	0.05	0.07
6	303,513	0.07	0.07	0.08
7	332,635	0.09	0.08	0.10
8	324,581	0.12	0.10	0.13
9	320,607	0.17	0.14	0.26
10	320,705	0.45	0.26	1.00

© 2021 Kipnis P et al. *JAMA Health Forum.*

eAPPENDIX. CALCULATION OF THE ABBREVIATED LABORATORY-BASED ACUTE PHYSIOLOGY SCORE (abLAPS)

Like the COmorbidity Point Score, version 2 (COPS2)[1], the abLAPS is assigned on a monthly basis to all Kaiser Permanente Northern California adults with a medical record number. The score is a variant of the LAPS score[2, 3] except that it uses a 1-month scoring time frame (patients' laboratory records are scanned for the preceding month). The score is used for internal predictive models in combination with the COPS2 because it permits distinguishing between stable and unstable patients. For example, a patient with a COPS2 of 102 is one with a significant comorbidity burden. However, a patient with a COPS2 of 102 and an abLAPS of 12 is a much sicker one than one with an abLAPS of 2. Neither the COPS2 or abLAPS are used as prediction tools directly; rather, they are scalars used in other predictive models or as population descriptors. The table below shows the laboratory tests employed to assign the score and the rule followed when multiple laboratory test results are available over the scoring period. Missing data are imputed to normal (abLAPS subscore = 0).

Laboratory test	Points	Test result to use if multiple tests in time frame
**Blood Urea Nitrogen (BUN)**		
< 18	0	Select HIGHEST
18 - 19	4	
20 - 39	12	
40 - 79	19	
80+	24	
**Creatinine**		
< 1.0	0	Select HIGHEST
1.0 - 1.9	1	
2.0 - 3.9	7	
4.0+	5	

© 2021 Kipnis P et al. *JAMA Health Forum.*

Laboratory test	Points	Test result to use if multiple tests in time frame
**BUN:Creatinine Ratio (*1000)**		
< 25.0	0	Select HIGHEST
25.0 +	6	
**Hematocrit**		
< 20.0	7	
20.0 - 39.9	5	
40.0 - 49.9	0	Select LOWEST
50.0 - 59.9	6	
60.0+	23	
**Arterial pH**		
< 7.15	30	
7.15 - 7.24	23	
7.25 - 7.34	16	
7.35 - 7.44	0	Select LOWEST
7.45 - 7.54	11	
7.55 - 7.64	14	
7.65+	14	
**Arterial PaCO2**		
< 25	5	
25 - 34	12	
35 – 44	0	Select HIGHEST
45 - 54	10	
55 - 64	9	
65+	13	

© 2021 Kipnis P et al. *JAMA Health Forum.*

Laboratory test	Points	Test result to use if multiple tests in time frame
**Arterial PaO2**		
< 50	13	
50 - 119	0	Select LOWEST
120+	18	
**Glucose**		
< 40	16	If there is a value < 60, select LOWEST<br><br>If there are no values < 60, select HIGHEST
40 - 59	12	
60 - 199	0	
200 - 349	3	
350+	3	
**Bilirubin**		
< 2.0	0	
2.0 - 2.9	10	
3.0 - 4.9	16	Select HIGHEST
5.0 - 7.9	22	
8.0+	32	
**Albumin**		
< 2.0	23	
2.0 - 2.4	18	Select LOWEST
2.5 - 4.4	0	
4.5+	0	
**White blood cell count**		
< 2.0	29	
2.0 - 4.9	6	Select LOWEST
5.0 - 12.9	0	
13.0+	15	

© 2021 Kipnis P et al. *JAMA Health Forum.*

Laboratory test	Points	Test result to use if multiple tests in time frame
**Troponin**		
0	0	Select HIGHEST
0.01 - 0.19	2	
0.20 - 0.99	6	
1.00 - 2.99	18	
3.00 - 5.99	20	
6.00+	25	
**Modified Sodium***		
0.0 – 4.9	0	Select HIGHEST
5.0 - 9.9	6	
10.0 - 15.9	7	
16.0 - 48.9	7	
49.0+	10	

* Algorithm to define **Modified Sodium**:

IF SODIUM = 135 to 145, Modified Sodium = 0;
ELSE IF SODIUM < 135, Modified Sodium = $[135 – SODIUM]^2$
ELSE Modified Sodium = $[SODIUM – 145]^2$

© 2021 Kipnis P et al. *JAMA Health Forum.*

eTable 6. Characteristics of Patients by Probability of COVID-19 Infection (PROVID) and COVID-19 Risk Score (CRS) Levels

PROVID/ CRS LEVEL	LOW/ LOW	LOW/ MED	LOW/ HIGH	MED/ LOW	MED/ MED	MED/ HIGH	HIGH/ LOW	HIGH/ MED	HIGH/ HIGH
N	271,322	391,202	618,792	492,840	296,371	171,763	499,065	286,139	175,943
AGE	30.5	53.3	70.9	30.7	49.3	64.8	29.9	49.4	64.7
Male	42%	37%	57%	41%	51%	61%	41%	50%	60%
Asian	27%	24%	18%	31%	29%	20%	8%	5%	4%
Black	0%	0%	2%	4%	9%	16%	12%	12%	14%
Hispanic	0%	0%	0%	0%	0%	6%	62%	70%	70%
White	69%	70%	73%	44%	42%	44%	7%	5%	8%
NDI†	-1.1	-0.9	-0.7	-0.3	-0.1	0.2	0.5	0.4	0.5
COPS2‡	9.9	9.9	20.8	9.8	10.0	35.4	9.8	10.0	34.4
COVID Hospitalizations per 10K	1.7	7.6	34.1	6.1	16.1	67.5	16.4	41.2	120.6
COVID-19 (+) per 1K	4.7	4.6	4.4	8.2	8.5	8.9	24.5	24.3	19.8
Deaths per 100K	0.4	2.0	44.6	0.8	5.4	89.1	1.4	17.1	146.6

†See text and Messer et al. (2006) for additional detail on the neighborhood deprivation index (NDI); this index ranges between -5 to +5, with more positive values indicating worsening neighborhood characteristics (e.g., poverty, unemployment). Number shown is median (interquartile range).

‡The COPS2 (COmorbidity Point Score, version 2), described in Escobar et al. (2013) is a score assigned every month to all adults with a Kaiser Permanente Northern California medical record number. Range is from 0 to 1010; higher scores indicate worse mortality risk. The univariate relationship between the COPS2 and 1-year mortality is as follows: 0-39, 0.3%; 40-64, 5.3%; 65+, 17.2%.

© 2021 Kipnis P et al. *JAMA Health Forum.*

eFigure 1. Estimated Avoidable Hospitalizations among Patients Vaccinated in First 8 Months by Vaccination Prioritization Order in Populations with Varying Age and Race Distributions

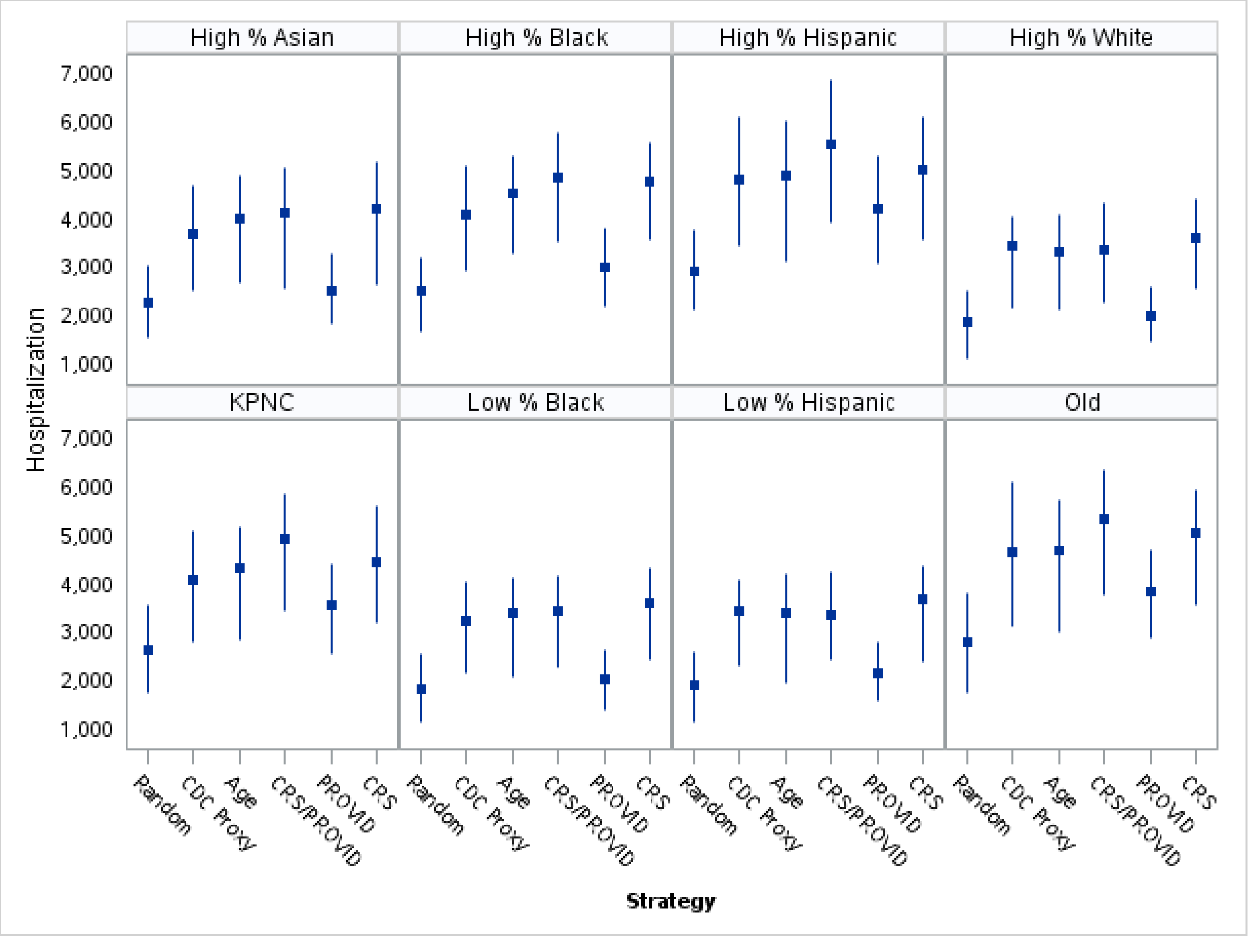

eFigure 2. Estimated Avoidable Household COVID-19 Deaths among Patients Vaccinated in First 8 Months by Vaccination Prioritization Order in Populations with Varying Age and Race Distributions

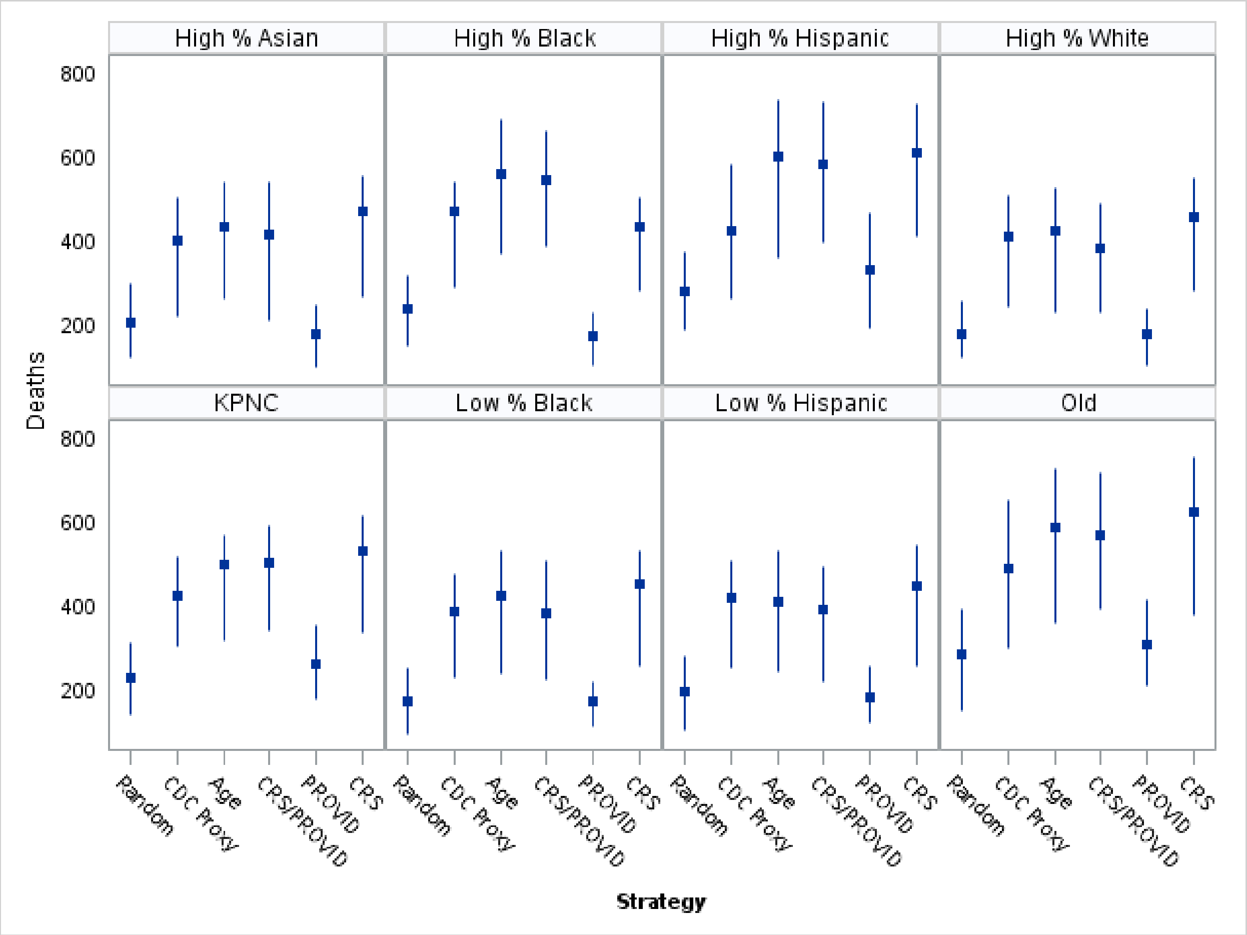

© 2021 Kipnis P et al. *JAMA Health Forum.*

eFigure 3. Estimated Avoidable Household COVID-19 Transmissions among Patients Vaccinated in First 8 Months by Vaccination Prioritization Order in Populations with Varying Age and Race Distributions

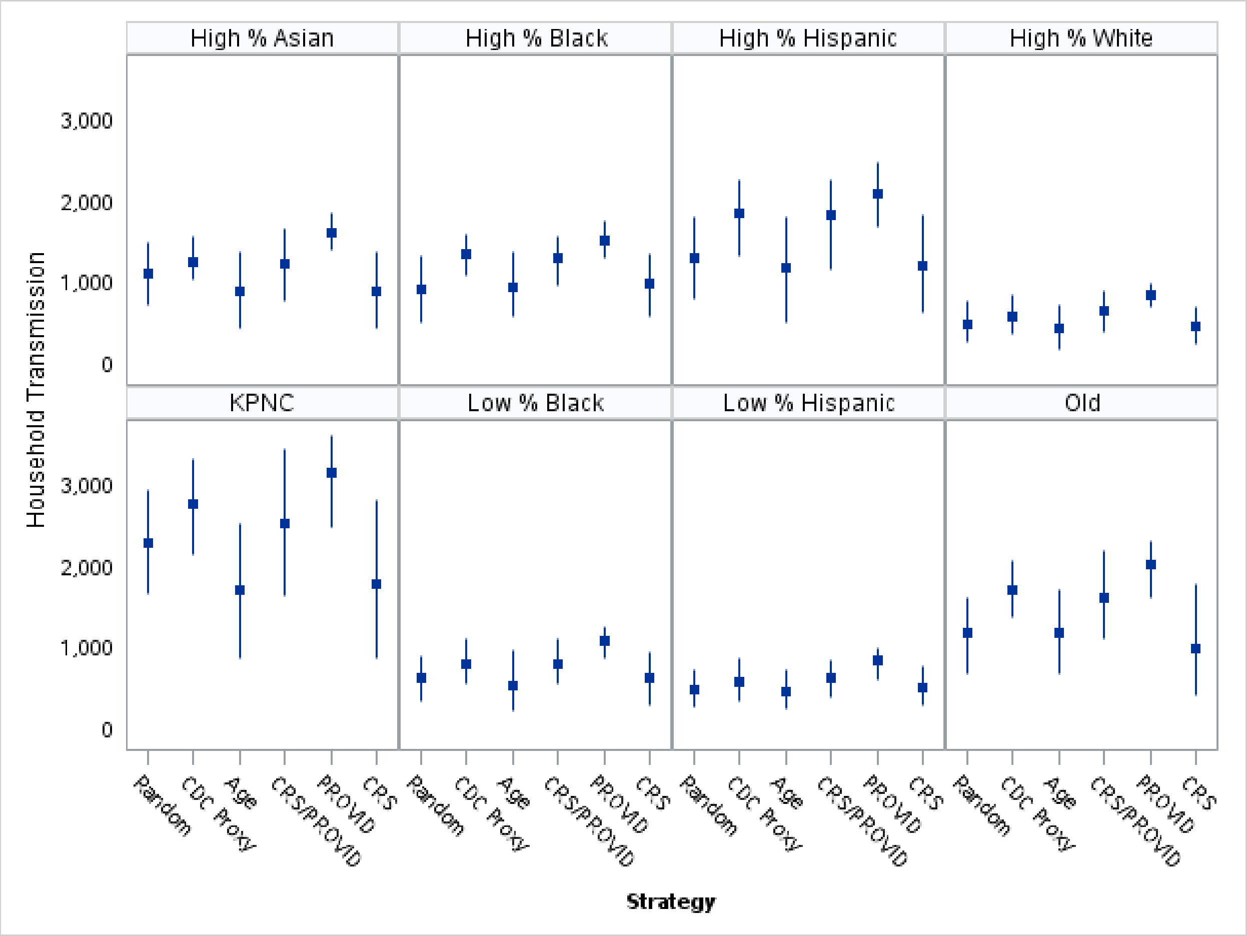

© 2021 Kipnis P et al. *JAMA Health Forum.*

eFigure 4. Cumulative Percent of Patients Vaccinated by Race, Vaccination Month and Strategy in Populations with Varying Age and Race Distributions

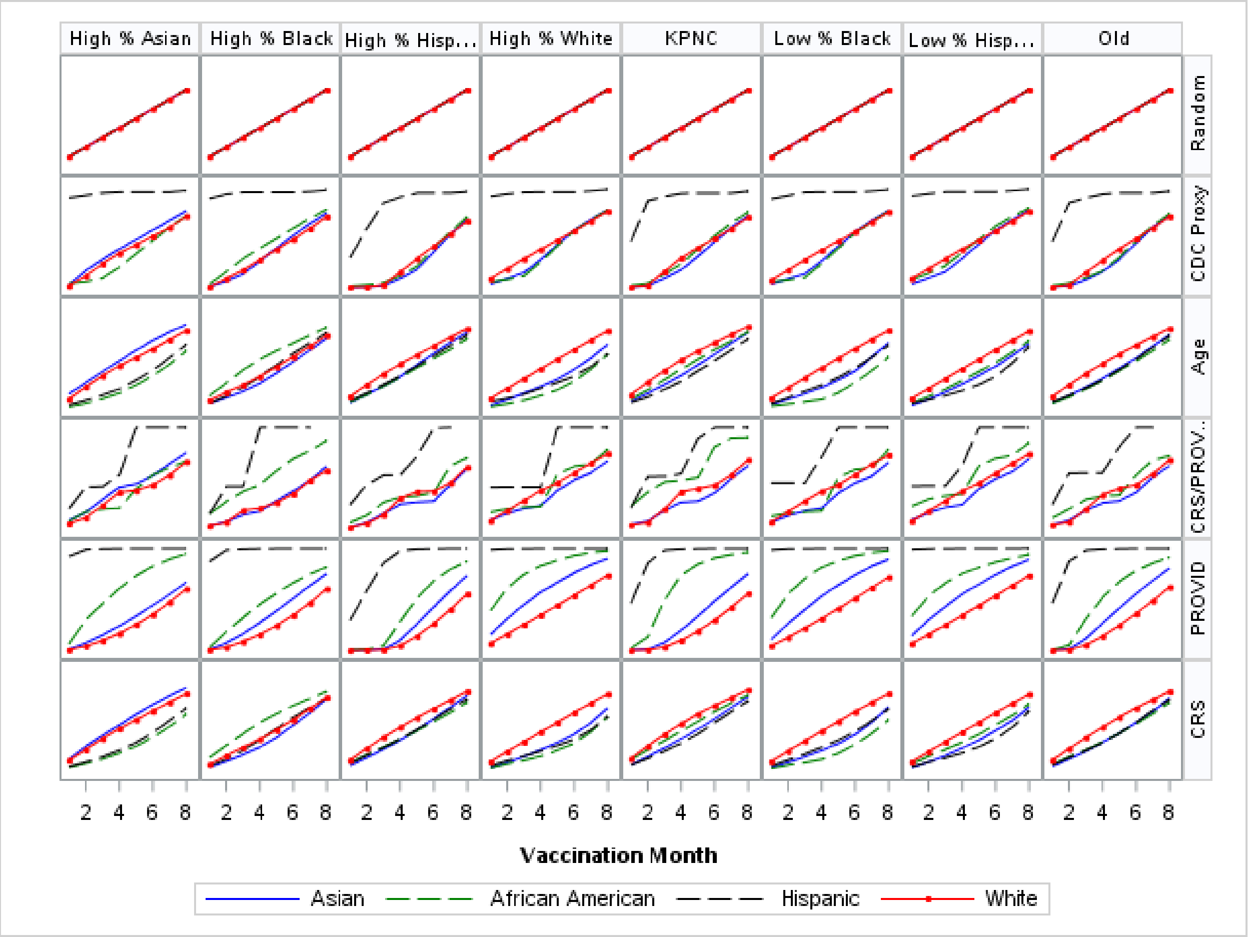

© 2021 Kipnis P et al. *JAMA Health Forum.*

eTable 7. Consolidated health economic evaluation reporting standards (CHEERS) statement checklist

Section/item	Item No	Recommendation	Reported on page No/ line No
**Title and abstract**			
Title	1	Identify the study as an economic evaluation or use more specific terms such as “cost-effectiveness analysis”, and describe the interventions compared.	Page 1
Abstract	2	Provide a structured summary of objectives, perspective, setting, methods (including study design and inputs), results (including base case and uncertainty analyses), and conclusions.	Pages 3-4
**Introduction**			
Background and objectives	3	Provide an explicit statement of the broader context for the study.	Page 5
		Present the study question and its relevance for health policy or practice decisions.	Page 5
**Methods**			
Target population and subgroups	4	Describe characteristics of the base case population and subgroups analyzed, including why they were chosen.	Page 6
Setting and location	5	State relevant aspects of the system(s) in which the decision(s) need(s) to be made.	Page 6
Study perspective	6	Describe the perspective of the study and relate this to the costs being evaluated.	Page 7
Comparators	7	Describe the interventions or strategies being compared and state why they were chosen.	Pages 8
Time horizon	8	State the time horizon(s) over which costs and consequences are being evaluated and say why appropriate.	Page 9
Discount rate	9	Report the choice of discount rate(s) used for costs and outcomes and say why appropriate.	NA
Choice of health outcomes	10	Describe what outcomes were used as the measure(s) of benefit in the evaluation and their relevance for the type of analysis performed.	Page 7
Measurement of effectiveness	11a	*Single study-based estimates:* Describe fully the design features of the single effectiveness study and why the single study was a sufficient source of clinical effectiveness data.	Pages 9-10
	11b	*Synthesis-based estimates:* Describe fully the methods used for identification of included studies and synthesis of clinical effectiveness data.	NA

© 2021 Kipnis P et al. *JAMA Health Forum.*

Measurement and valuation of preference based outcomes	12	If applicable, describe the population and methods used to elicit preferences for outcomes.	NA
Estimating resources and costs	13a	*Single study-based economic evaluation:* Describe approaches used to estimate resource use associated with the alternative interventions. Describe primary or secondary research methods for valuing each resource item in terms of its unit cost. Describe any adjustments made to approximate to opportunity costs.	Pages 9-10
	13b	*Model-based economic evaluation:* Describe approaches and data sources used to estimate resource use associated with model health states. Describe primary or secondary research methods for valuing each resource item in terms of its unit cost. Describe any adjustments made to approximate to opportunity costs.	NA
Currency, price date, and conversion	14	Report the dates of the estimated resource quantities and unit costs. Describe methods for adjusting estimated unit costs to the year of reported costs if necessary. Describe methods for converting costs into a common currency base and the exchange rate.	NA
Choice of model	15	Describe and give reasons for the specific type of decision-analytical model used. Providing a figure to show model structure is strongly recommended.	Pages 9-10
Assumptions	16	Describe all structural or other assumptions underpinning the decision-analytical model.	Page 8: lines 153, 156, 158<br>Page 9: lines 185, 193<br>Page 14: lines 322 & 325
Analytical methods	17	Describe all analytical methods supporting the evaluation. This could include methods for dealing with skewed, missing, or censored data; extrapolation methods; methods for pooling data; approaches to validate or make adjustments (such as half cycle corrections) to a model; and methods for handling population heterogeneity and uncertainty.	Pages 9-10
**Results**			
Study parameters	18	Report the values, ranges, references, and, if used, probability distributions for all parameters. Report reasons or sources for distributions used to	Page 10, Table 1 and Appendix A

© 2021 Kipnis P et al. *JAMA Health Forum.*

		represent uncertainty where appropriate. Providing a table to show the input values is strongly recommended.	
Incremental costs and outcomes	19	For each intervention, report mean values for the main categories of estimated costs and outcomes of interest, as well as mean differences between the comparator groups. If applicable, report incremental cost-effectiveness ratios.	Figures 1, 2, 3
Characterizing uncertainty	20a	*Single study-based economic evaluation:* Describe the effects of sampling uncertainty for the estimated incremental cost and incremental effectiveness parameters, together with the impact of methodological assumptions (such as discount rate, study perspective).	All results show confidence intervals. The sensitivity analysis addressed the uncertainty in input parameters.
	20b	*Model-based economic evaluation:* Describe the effects on the results of uncertainty for all input parameters, and uncertainty related to the structure of the model and assumptions.	
Characterizing heterogeneity	21	If applicable, report differences in costs, outcomes, or cost-effectiveness that can be explained by variations between subgroups of patients with different baseline characteristics or other observed variability in effects that are not reducible by more information.	Page 11, Lines 235-252
Study findings, limitations, generalizability, and current knowledge	22	Summarize key study findings and describe how they support the conclusions reached. Discuss limitations and the generalizability of the findings and how the findings fit with current knowledge.	Page 14-15
**Other**			
Source of funding	23	Describe how the study was funded and the role of the funder in the identification, design, conduct, and reporting of the analysis. Describe other non-monetary sources of support.	Page 15
Conflicts of interest	24	Describe any potential for conflict of interest of study contributors in accordance with journal policy. In the absence of a journal policy, we recommend authors comply with International Committee of Medical Journal Editors recommendations.	Page 15

© 2021 Kipnis P et al. *JAMA Health Forum.*

eTable 8. Age and Race/Ethnicity Distribution across U.S. States[4]

State	Population	65+	Asian	Black	Hispanic	White
Alabama	4,876,250	21.3%	1.3%	26.8%	4.5%	65.1%
Alaska	737,068	14.9%	5.9%	3.0%	7.2%	59.8%
Arizona	7,050,299	22.3%	3.2%	4.4%	31.7%	54.0%
Arkansas	2,999,370	21.8%	1.5%	15.4%	7.7%	72.0%
California	39,283,497	18.2%	14.6%	5.5%	39.4%	36.3%
Colorado	5,610,349	17.8%	3.2%	4.0%	21.8%	67.5%
Connecticut	3,575,074	21.2%	4.6%	10.1%	16.9%	65.6%
Delaware	957,248	23.1%	3.7%	22.0%	9.6%	61.3%
District of Columbia	692,683	14.7%	4.0%	44.1%	11.3%	37.3%
Florida	20,901,636	25.1%	2.7%	15.2%	26.4%	53.0%
Georgia	10,403,847	17.8%	4.1%	31.5%	9.8%	51.8%
Hawaii	1,422,094	22.6%	38.1%	1.8%	10.7%	21.5%
Idaho	1,717,750	20.7%	1.4%	0.7%	12.8%	81.6%
Illinois	12,770,631	19.6%	5.6%	13.9%	17.5%	60.7%
Indiana	6,665,703	20.2%	2.5%	9.4%	7.2%	78.3%
Iowa	3,139,508	21.7%	2.4%	4.0%	6.3%	85.1%
Kansas	2,910,652	20.4%	2.9%	5.5%	12.2%	75.4%
Kentucky	4,449,052	20.6%	1.6%	8.0%	3.8%	84.2%
Louisiana	4,664,362	19.5%	1.7%	32.1%	5.4%	58.2%
Maine	1,335,492	24.7%	1.1%	1.5%	1.7%	92.8%
Maryland	6,018,848	19.3%	6.3%	29.7%	10.6%	49.8%
Massachusetts	6,850,553	20.1%	6.8%	7.1%	12.4%	70.3%
Michigan	9,965,265	21.5%	3.3%	13.5%	5.3%	74.7%
Minnesota	5,563,378	20.1%	5.0%	6.4%	5.6%	78.9%
Mississippi	2,984,418	20.4%	1.0%	37.8%	3.0%	56.3%
Missouri	6,104,910	21.3%	2.1%	11.4%	4.3%	79.1%
Montana	1,050,649	23.1%	0.8%	0.6%	3.8%	85.8%
Nebraska	1,914,571	20.3%	2.4%	4.8%	11.3%	78.4%
Nevada	2,972,382	20.0%	8.3%	9.3%	29.2%	47.8%
New Hampshire	1,348,124	21.7%	2.6%	1.4%	4.0%	89.7%
New Jersey	8,878,503	20.3%	9.6%	12.7%	20.9%	54.3%
New Mexico	2,092,454	22.0%	1.6%	1.9%	49.3%	36.8%
New York	19,572,319	20.4%	8.6%	14.2%	19.3%	55.1%
North Carolina	10,264,876	20.4%	2.9%	21.1%	9.8%	62.5%
North Dakota	756,717	19.4%	1.4%	2.9%	4.0%	83.6%
Ohio	11,655,397	21.5%	2.3%	12.4%	4.0%	78.3%
Oklahoma	3,932,870	20.3%	2.2%	7.1%	11.1%	64.9%
Oregon	4,129,803	21.6%	4.5%	1.8%	13.4%	74.9%
Pennsylvania	12,791,530	22.5%	3.5%	10.7%	7.8%	75.6%
Rhode Island	1,057,231	24.5%	3.4%	5.8%	16.3%	70.8%
South Carolina	5,020,806	21.0%	1.7%	26.3%	5.8%	63.5%

© 2021 Kipnis P et al. *JAMA Health Forum.*

State	Population	65+	Asian	Black	Hispanic	White
South Dakota	870,638	22.1%	1.3%	2.3%	3.7%	81.5%
Tennessee	6,709,356	21.6%	1.8%	16.6%	5.7%	73.3%
Texas	28,260,856	20.6%	4.9%	11.9%	39.7%	41.1%
Utah	3,096,848	16.6%	2.4%	1.1%	14.4%	77.7%
Vermont	624,313	15.5%	1.4%	1.3%	2.0%	92.5%
Virginia	8,454,463	23.1%	6.6%	19.0%	9.7%	61.1%
Washington	7,404,107	19.4%	8.9%	3.9%	13.0%	67.3%
West Virginia	1,817,305	19.4%	0.8%	3.6%	1.5%	92.0%
Wisconsin	5,790,716	24.3%	2.9%	6.3%	7.1%	80.8%
Wyoming	581,024	21.2%	0.8%	1.1%	10.1%	83.7%
Puerto Rico	3,318,447	20.5%	0.1%	0.1%	98.2%	1.3%

REFERENCES

1. Escobar GJ, Gardner MN, Greene JD, Draper D, Kipnis P. Risk-adjusting hospital mortality using a comprehensive electronic record in an integrated health care delivery system. *Med Care*. May 2013;51(5):446-53. doi:10.1097/MLR.0b013e3182881c8e
2. Escobar G, Greene J, Scheirer P, Gardner M, Draper D, Kipnis P. Risk Adjusting Hospital Inpatient Mortality Using Automated Inpatient, Outpatient, and Laboratory Databases. *Medical Care*. March 2008;46(3):232-39.
3. van Walraven C, Escobar GJ, Greene JD, Forster AJ. The Kaiser Permanente inpatient risk adjustment methodology was valid in an external patient population. *J Clin Epidemiol*. July 2010;63(7):798-803.
4. Bureau USC. American Community Survey, 2018; American Community Survey 5-Year Estimates, Table B03002. Accessed May 31, 2021, 2021. https://data.census.gov/cedsi/ Accessed June 1, 2020

© 2021 Kipnis P et al. *JAMA Health Forum.*
